# Increased Expression of Syncytin-1 in Skeletal Muscle of Humans With Increased Body Mass Index

**DOI:** 10.3389/fphys.2022.858341

**Published:** 2022-04-04

**Authors:** Jayachandran Ravichandran, Lori R. Roust, Christos S. Katsanos

**Affiliations:** ^1^ School of Life Sciences, Arizona State University, Tempe, AZ, United States; ^2^ College of Medicine, Mayo Clinic in Arizona, Scottsdale, AZ, United States; ^3^ Department of Physiology and Biomedical Engineering, Mayo Clinic in Arizona, Scottsdale, AZ, United States

**Keywords:** syncytin-1, obesity, muscle, protein synthesis, fatty acids, insulin

## Abstract

Obesity negatively impacts skeletal muscle protein metabolism, and also impairs skeletal muscle maintenance and regeneration. We analyzed muscle biopsy samples from humans with increased body mass index (BMI) (i.e. > 30 kg/m^2^) and controls (i.e., BMI < 25 kg/m^2^) for expression of syncytin-1, a fusogenic protein regulating skeletal muscle regeneration. When compared to controls, humans with increased BMI and concomitant reduction in muscle protein synthesis had higher expression of syncytin-1 in skeletal muscle (*p* < 0.05). Across human subjects, muscle protein synthesis correlated inversely (*r* = −0.51; *p* = 0.03) with syncytin-1 expression in muscle. Using a C2C12 cell line we found that expression of syncytin-A (i.e, corresponding protein in murine tissue) is increased by insulin, and that this response is impaired in the presence of fatty acids, whose metabolism is altered within the metabolic environment induced by increased BMI. In C2C12 cells, the response of the protein 4E-BP1, which signals increase in protein synthesis in muscle, resembled that of syncytin-A. These findings provide novel insights into the expression of syncytin-1 in skeletal muscle of humans with increased BMI, as well as its basic regulation by insulin and fatty acids in muscle. The findings signify the need for further research into the regulation of syncytin-1 in skeletal muscle of humans with increased BMI, as well as its biological implications for altering muscle protein metabolism and regeneration.

## Introduction

Understanding the biological mechanisms that alter protein metabolism and tissue growth in skeletal muscle under various pathophysiological circumstances constitutes currently an active area of research. Protein synthesis is reported lower in muscle of humans characterized by body mass index (BMI) > 30 kg/m^2^ (i.e., humans with obesity) (Guillet et al., 2009; Bak et al., 2016; Tran et al., 2016; Tran et al., 2018), which provides a mechanism that may reduce muscle mass in these individuals. This phenomenon is evident in clinical circumstances, and described as “sarcopenic obesity”. Moreover, obesity may impair skeletal muscle maintenance and regeneration possibly due to compromised skeletal muscle cell fusion (Akhmedov and Berdeaux, 2013). The mechanisms that impair muscle protein synthesis as well as muscle maintenance and regeneration in humans with increased BMI remain currently elusive, limiting our understanding of the effects of obesity on muscle protein metabolism and growth.

The protein syncytin-1, encoded by the human endogenous retrovirus group W envelope member 1 (HERVW-1) gene, is responsible for cell fusion, and its role has been characterized to date largely in placental development (Lokossou et al., 2014). In murine tissue, this function is accomplished by syncytin-A. Phylogenetic analysis shows that syncytin-A and syncytin-1 fall under the same HERV family, with syncytin-A being homologous to the human syncytin gene, and that syncytin-A and syncytin-1 carry out the same fusogenic functions (Dupressoir et al., 2005). Although most research to date has focused on the role of syncystin-1 on placental tissue, syncytin-1 is present also in skeletal muscle, and specifically in the sarcolemma of the muscle fibers (Frese et al., 2015), where it regulates cell fusion in muscle (Oluwole et al., 2007; Bjerregaard et al., 2011; Frese et al., 2015). Given that syncytin-1 has key role in myogenesis (Frese et al., 2015), a process that is impaired in humans with obesity (Akhmedov and Berdeaux, 2013), investigating the expression of syncytin-1 in muscle of humans with increased BMI may provide novel insights into the development of sarcopenia in such individuals.

There is very limited evidence describing syncytin-1 expression in skeletal muscle of humans (Oluwole et al., 2007; Frese et al., 2015), and there is no evidence describing syncytin-1 expression specifically in the muscle of humans with increased BMI. Also, there is paucity of evidence about biological signals regulating the expression of syncytin-1 in muscle. Relevant biological signals affected in the metabolic context of obesity and associated insulin resistance include the insulin and fatty acids. Although total plasma free fatty acid concentrations are not necessarily higher in humans with obesity (Karpe et al., 2011), obesity is associated with skeletal muscle lipotoxicity that affects protein metabolism in muscle (Meex et al., 2019). In this regard, effects of fatty acids on impairing muscle metabolism are linked to specific fatty acids species, with palmitate being detrimental for myogenesis (da Paixão et al., 2021) and overall muscle protein anabolism (Tardif et al., 2011), whereas oleate opposes the detrimental effects of palmitate on protein metabolism in muscle (Lee et al., 2017). Experiments in C2C12 cells show that palmitate, but not oleate, impairs signaling for protein synthesis in skeletal muscle, documented as decreased activation of the eukaryotic translation initiation factor 4E (eIF4E)-binding protein 1 (4E-BP1) (Kwon and Querfurth, 2015).

Given the role of syncytin-1 in myogenesis, we tested the hypothesis that humans with increased BMI and concomitant reduction in skeletal muscle protein synthesis have lower expression of syncytin-1 in skeletal muscle. Moreover, we evaluated the effects of biological signals known to be altered in the metabolic environment of obesity on muscle syncytin-A expression in cell culture. We hypothesized that insulin stimulates syncytin-A expression and that this effect is not evident in the presence of fatty acids.

## Materials and Methods

### Human Subjects

We evaluated syncytin-1 expression in muscle biopsy samples from humans with BMI > 30 kg/m^2^ and control humans with BMI < 25 kg/m^2^. Muscle samples analyzed were collected in a previous study where we found that humans with BMI > 30 kg/m^2^ have lower muscle protein synthesis compared to that of control subjects with BMI < 25 kg/m^2^ (Tran et al., 2018). Study participants were recruited through flyer advertisements from the greater Phoenix Metro area, and the campuses of Arizona State University and Mayo Clinic in Arizona. Study exclusion criteria included medication or supplements known to affect protein metabolism (i.e. amino acids, protein, fish oil), presence of acute illness, liver disease, renal disease, heart disease, clinically abnormal hemoglobin or hematocrit values, Diabetes, current participation in a weight-loss program, extreme dietary practices, smoking, and use of anabolic steroids or corticosteroids within the last 3 months. Limited evidence describing syncytin-1 gene expression in skeletal muscle of healthy humans (Frese et al., 2015) suggests an effect size of approximately 1.5 in association with a change in syncytin-1 gene expression in muscle. Statistical power calculations (Faul et al., 2007) indicated that for a standard power of 80% and α error of 0.05, approximately eight subjects per group can detect a difference corresponding to a comparable effect size (i.e., 1.5) between subjects with increased BMI and controls subjects. The study procedures were approved by the Institutional Review Board at Mayo Clinic. Research took place in the Clinical Studies Infusion Unit (CSIU) at Mayo Clinic in Arizona, Scottsdale campus.

### Screening of the Subjects

Subjects arrived in the CSIU in the morning after an overnight fasting period (∼10 h). Subjects had a blood draw to determine fasting blood chemistry parameters and then underwent a 2 h oral glucose tolerance test (OGTT). Subjects with evidence of diabetes (i.e., fasting plasma glucose ≥ 126 mg/dl or 2 h plasma glucose during the OGTT ≥ 200 mg/dl) were excluded from the study. Body composition was determined using bioelectrical impedance analysis (BIA; BIA 310e, Biodynamics Corp., Shoreline, Washington). All subjects were asked to follow specific instructions prior to the BIA to improve measurements of body composition (i.e., no caffeine, food, or alcohol for > 10–12 h; arrive well-hydrated) (Utter et al., 1999; Chittawatanarat et al., 2011). Waist and hip circumference measurements were performed using procedures previously described (WHO, 2011). Table 1 displays the characteristics of the two subject populations. Participants in the group of subjects with increased BMI identified as White (*n* = 10) (2 Hispanic) and those in the control group identified as White (*n* = 7) and Asian (*n* = 1) (1 Hispanic).

**TABLE 1:** Subject characteristics.

	BMI < 25 kg/m^2^	BMI> 30 kg/m^2^
n (F/M)	8 (5/3)	10 (4/6)
Age (years)	34.5 ± 10.8	36.3 ± 8.8
Weight (kg)	65.0 ± 13.5	101.9 ± 14.6*
BMI (kg/m^2^)	22.4 ± 2.7	34.4 ± 3.3*
Waist (cm)	79.9 ± 7.4	106.9 ± 10.8*
Waist-to-hip ratio	0.80 ± 0.04	0.91 ± 0.09*
FFM (kg)	49.5 ± 11.3	67.8 ± 9.7*
Body fat mass (%)	23.9 ± 7.3	33.1 ± 7.5*
Fasting plasma glucose (mg·dl^−1^)	85.6 ± 6.7	98.4 ± 13.9*
Fasting plasma insulin (uIU·ml^−1^)	3.6 ± 0.8	10.9 ± 6.0*
HOMA-IR	0.8 ± 0.2	2.7 ± 1.8*
Matsuda-ISI	9.4 ± 2.2	4.6 ± 4.3*
HbA1c (%)	5.4 ± 0.3	5.7 ± 0.4*
Plasma triglycerides (mg·dl^−1^)	70.9 ± 28.3	191.0 ± 140.8*
Plasma total cholesterol (mg·dl^−1^)	174.1 ± 34.6	178.8 ± 32.2
Plasma HDL-Cholesterol (mg·dl^−1^)	71.5 ± 18.4	40.9 ± 8.8*
HDL-Cholesterol:Total Cholesterol	0.4 ± 0.1	0.2 ± 0.1*
Plasma LDL-Cholesterol (mg·dl^−1^)	88.4 ± 28.3	98.4 ± 21.6

Values are mean ± SD. BMI, body mass index; FFM, fat-free mass; HOMA-IR, homeostatic model assessment of insulin-resistance; Matsuda-ISI, Matsuda insulin-sensitivity index (as discussed in text); HbA1c, glycated hemoglobin; HDL, high-density lipoprotein; LDL, low-density lipoprotein; *p < 0.05 versus subjects with BMI < 25 kg/m^2^.

### Stable Isotope Infusion Experiments and Muscle Biopsies

A catheter was placed into an antecubital arm vein for infusion (15 μmol kg FFM^−1^ min^−1^; priming dose, 6.4 μmol kg FFM^−1^) of d_10_-leucine (L-[2,3,3,4,5,5,5,6,6,6-^2^H_10_]leucine) to measure rate of muscle protein synthesis, while another catheter was placed in a retrograde fashion in a dorsal hand vein for blood sampling. A percutaneous muscle biopsy (∼100 mg) of the vastus lateralis was obtained with a Bergström biopsy needle under local anesthesia (lidocaine, 2%) at 120 and 300 min after the start of the d_10_-leucine infusion, and while subjects remained resting in bed. The muscle sample was rinsed with cold saline to remove blood, blotted dry, and cleaned of any visible fat and connective tissue before placing in liquid nitrogen for later analysis. Blood samples were collected at 110, 115, 140, 260, 280, and 300 for determining blood d_9_-leucine enrichment.

### Muscle Protein Synthesis

Leucine enrichments in the muscle and blood samples were used to quantify protein fractional synthesis rate in skeletal muscle, and by following procedures we have previously described (Tran et al., 2015). Briefly, blood samples were collected in tubes containing 15% sulfosalicylic acid (SSA), and mixed well prior to centrifugation to collect the supernatant. The supernatant was passed through cation-exchange columns (AG 50W-8x 100–200-mesh; Bio-Rad Laboratories, Inc.) to isolate the blood amino acids, and which were eluted using 8 ml of 2N NH4OH. For muscle, ∼15 mg of muscle was homogenized in the presence of 0.5 ml of 5% sulfosalicylic acid to precipitate the muscle proteins. The proteins were hydrolyzed with 6 N HCl at 110°C for 24 h. This sample was passed through cation-exchange column (AG 50W-8x 200–400-mesh; Bio-Rad Laboratories, Inc.) to isolate/purify the amino acids, and which were eluted from the columns with of 2N NH4OH. Isotopic enrichment of amino acids with d_9_-leucine was measured using liquid chromatography tandem mass spectrometry (LC/MS/MS), and by following procedures we have also previously described (Tran et al., 2015). Fractional synthesis rate (FSR; %/hour) of muscle protein was calculated as:

$$\text{FSR}=\frac{\Delta \text{Em}}{\text{Eb}}\text{ x }60\text{ x }100$$

Where ΔEm is the increment in muscle protein leucine enrichment between the two biopsies, Eb is the average leucine enrichment in blood between the biopsies, and T is the time interval (i.e., in mins) between the biopsies (60 and 100 are used as factors to express the FSR values in %/hour).

### Plasma Chemistry Parameters

Plasma glucose concentrations were measured using an automated glucose analyzer (YSI 2300, Yellow Springs, OH). Plasma insulin concentrations were measured using a commercially available ELISA kit (80-INSHU-E01.1; ALPCO Diagnostics, Windham, NH). Plasma glucose and insulin concentrations during the OGTT were used to calculate the Matsuda insulin-sensitivity index (Matsuda and DeFronzo, 1999). The rest of the blood chemistry parameters reported in Table 1 were measured by the Mayo Clinic Clinical Laboratory. The concentration of major species of free fatty acids in plasma (arachidonic acid, elaidic acid, linoleic acid, linolenic acid, myristic acid, oleic acid, palmitic acid, palmitoleic acid, stearic acid) was measured by LC/MS (Persson et al., 2010).

### Cell Culture Experiments

Using a C2C12 cell culture model we sought to evaluate how biological signals altered within the metabolic environment of obesity affect syncytin-A expression in muscle. This experimental model allows isolating specific effects on syncytin-A expression in muscle, and in the absence of other concurrent biological signals present *in vivo* in humans. Insulin is well-established as a muscle anabolic hormone. Current evidence indicates specific roles of palmitate and oleate in regulating muscle growth and protein metabolism (da Paixão et al., 2021; Tardif et al., 2011; Lee et al., 2017; Kwon and Querfurth, 2015). Also, we found differential contribution of these fatty acid species to the total plasma free fatty acid concentrations in our human subjects with increased BMI (see Results section). Therefore, we chose to specifically test the effects of palmitate and oleate in our cell culture experiments.

C2C12 myoblasts (RRID:CVCL_0188) obtained from American Type Culture Collection (ATCC; Cat#: CRL-1772) were used for the cell culture experiments. C2C12 myoblasts were grown in growth media containing Dulbecco’s Modified Eagle’s Medium (DMEM) with 20% fetal bovine serum and 1% antibiotic-antimycotic at 37°C. The cells were grown in 6-well Collagen I-coated plates (Thermo Fisher Scientific; Cat #: A1142801). After 95% confluency, cells were differentiated in differentiation media containing DMEM with 2% horse serum and 1% antibiotic-antimycotic for 5 days, and until the cells were spindle-shaped (Kwon and Querfurth, 2015).

A 200 mM stock solution of palmitate and oleate were prepared using sodium oleate and palmitate dissolved in 50% ethanol for 30 min at 70°C. A 10% solution of fatty acid-free bovine serum albumin (BSA) was prepared in phosphate-buffered saline for conjugation purposes. Then, 5 mM stock solution of BSA conjugated fatty acid solutions (i.e., palmitate and oleate) were prepared by adding 200 mM stock solution of oleate and palmitate in 10% fatty acid-free BSA. Conjugation of the fatty acids and BSA was performed at 37°C for 1 h before cell treatment. C2C12 cells were incubated in serum free DMEM media for 2 h before treatment. The conjugated fatty acid-BSA serum was filtered and added to serum free DMEM media. Cells were treated with either 300 μm palmitate, 300 μm oleate, their combination, and with or without 20 nM insulin for 24 h. Before harvesting, cells were stimulated with 100 nM of insulin for 15 min. Myotubes were harvested as described by Kwon and Querfurth (Kwon and Querfurth, 2015).

### Immunoblotting

Lysates of human muscle and C2C12 myotubes were prepared following procedures we have previously used in our laboratory (Tran et al., 2016). Protein concentration in the lysates was measured using Pierce™ Coomassie Plus (Bradford) protein assay reagent kit (ThermoFisher Scientific; Cat# 23200), and by following the manufacturer’s protocol. Briefly, BSA standards were used in the concentrations of 0, 0.2, 0.4, 0.6, 0.8, and 1 mg for standard curve measurements. Lysates were diluted in a ratio of 1:40 (Lysate: Mili Q water). A 10 μl of the standard or the lysate was loaded into a 96 well plate. 190 μl of the Coomassie/Bradford reagent was added and the plate was incubated at room temperature for 10 min before measuring the absorbance at 595 nm in a spectrophotometer. Approximately 40 ug of protein from the human muscle lysate and 15 ug of protein from the myotube lysate was diluted (1:1) in a 2X Laemmli sample buffer and boiled for 5 min at 95°C, and the proteins were separated by SDS-PAGE on Any kD™ precast polyacrylamide gels (Mini-PROTEAN TGX, Bio-Rad Laboratories, Inc.). Proteins were transferred to nitrocellulose membrane in the case of human muscle homogenates and polyvinylidene difluoride membrane in case of C2C12 cell lysates for 1 h at 90 V, followed by incubation of the membrane with primary antibodies overnight at 4°C. Prior to incubation, the membranes were blocked at room temperature with TBST +5% non-fat dry milk (Syncytin-1/A) or TBST +5% BSA (4E-BP1, p-4E-BP1) for 60 min.

Syncytin-1 expression was quantified using an anti-syncytin-1 primary antibody (Biorbyt; Cat# orb100573; RRID:AB_2857960). The same antibody was used also in the C2C12 myotube experiments. Although the immunogen sequence was designed to detect human syncytin-1, it allows for mouse cross-reactivity and detection of syncytin-A in a mouse cell line (Ethiraj et al., 2018). Other primary antibodies used were: anti-GAPDH (Rockland Immunochemicals; Cat# 600-401-A33; RRID:AB_2107593), anti-4E-BP1 (Cell Signaling Technology; Cat# 9452; RRID:AB_331692), anti-phospho-4E-BP1 (Thr^37/46^) (Cell Signaling Technology; Cat# 9459; RRID:AB_330985), and anti-myosin (Santa Cruz Biotechnology; Cat# sc-32732; RRID:AB_670118). A dilution of 1:1000 was used for all primary antibodies in Tris-buffered saline with 0.1% Tween®-20 (TBST) + 5% BSA, except for that for syncytin-1, which was diluted at 1:250 in TBST. We chose myosin (instead of GAPDH) as the housekeeping protein for the cell culture experiments because GAPDH showed considerable variation across the cell culture experimental conditions (see Results section).

The following day, the membrane was washed with TBST and incubated for 60 min at room temperature with secondary antibody. Secondary antibodies used were anti-rabbit (Cat. # 205718; Abcam) and anti-mouse (Cat. # sc-516102; Santa Cruz Biotechnology) IgG HRP-linked antibodies at a dilution of 1:2000 (anti-mouse) or 1:5000 (anti-rabbit) in TBST. Excess secondary antibody was washed with TBST.

Protein bands were visualized using the Clarity™ Western ECL Blotting Substrate (Bio-Rad, Hershey, PA) and imaged using the ImageQuant LAS 4000 (GE Healthcare, Wauwatosa, WI). Images of the membranes were captured in the increasing exposure intervals of 30 s except for GAPDH which was imaged at 5 s. Density of bands was quantified using the ImageJ software (National Institutes of Health, Bethesda, MD). The band intensity of the protein of interest was normalized against the respective band intensity of the housekeeping protein present in the same lane.

### Statistical Analyses

Unpaired *t*-test or one-way ANOVA with Dunnett’s post-hoc tests were employed to compare data from two or more than two experimental groups, respectively. A two-way ANOVA with Bonferroni correction for multiple comparisons was used for two-factor analysis (i.e., BMI x sex). Correlations were evaluated using the Pearson product-moment correlation coefficient (*r*). Data are presented as means ± SD. *p* value of <0.05 was considered statistically significant. All statistical tests were two-sided. Statistical analyses were performed using GraphPad Prism version 8.4 (GraphPad Software, La Jolla, CA).

## Results

### Subject Characteristics

Subject characteristics shown in Table 1 indicate that the two subject groups represent distinct populations in terms of body composition and metabolic characteristics. In addition to having greater percent body fat, subjects with increased BMI were also characterized by significantly higher waist-to-hip ratio, and increased plasma insulin, glucose and triglyceride concentrations, as well as insulin resistance, all typical observations in humans with obesity.

Although the total concentration of the measured plasma free fatty acids did not differ between subjects with increased BMI and controls, plasma oleate concentrations were lower in the group with increased BMI (Supplementary Table S1). Plasma oleate and palmitate concentrations constituted ∼62% of the total plasma free fatty acids measured, with palmitic acid contributing significantly more (27.6 ± 1.7 versus 25.9 ± 1.1%; *p* = 0.02) and oleic acid contributing significantly less (33.4 ± 2.0 versus 37.2 ± 2.4%; *p* < 0.01) to the total plasma free fatty acids in the subjects with increased BMI compared to controls.

### Syncytin-1 Expression in Human Subjects

**FIGURE 1:**
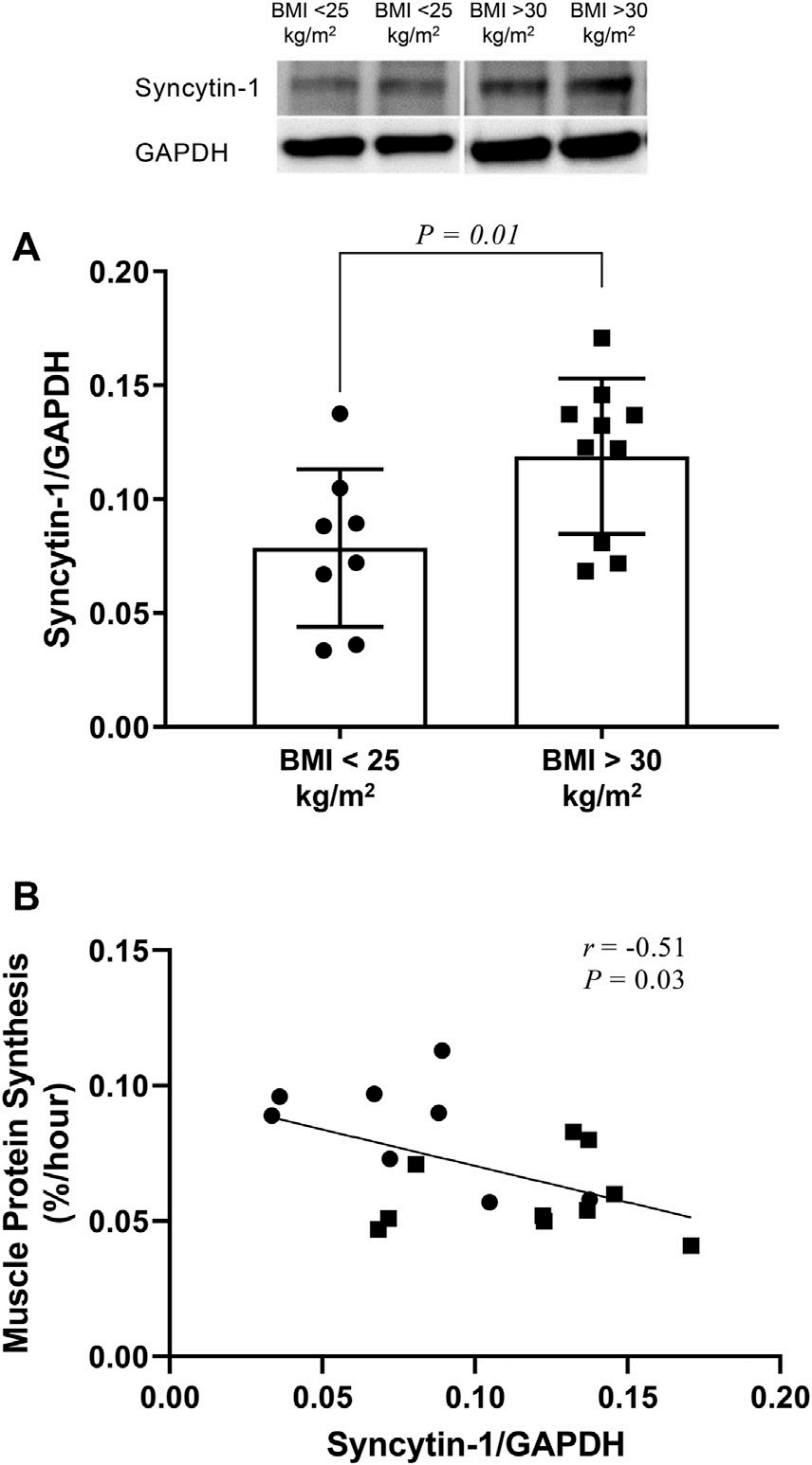
Western blot analysis of syncytin-1 expression in skeletal muscle of subjects with increased BMI (i.e,. BMI > 30 kg/m^2^) and control subjects (i.e., BMI < 25 kg/m^2^). Individual data points are shown along with mean ± SD **(A)**. Pearson product-moment correlation (r) between syncytin-1 expression and protein synthesis in skeletal muscle across all subjects whose data are depicted in panel A above **(B)**.

When compared to that in control subjects, skeletal muscle syncytin-1 expression was higher in the group of subjects with increased BMI and concomitant reduction in muscle protein synthesis (protein synthesis: 0.059 ± 0.014 versus 0.084 ± 0.019%/hour; *p* = 0.01) (Figure 1A). ANOVA analysis indicated no overall effect for sex (*p* = 0.54). Interestingly, although it did not reach statistical significance (probably because of low sample size), female (0.134 ± 0.038 versus 0.078 ± 0.027; *p* = 0.07), but not male (0.109 ± 0.031 versus 0.080 ± 0.052; *p* = 0.32), subjects with increased BMI displayed increased expression of syncytin-1 in skeletal muscle.

Expression of syncytin-1 in muscle across study subjects correlated significantly and inversely with muscle protein synthesis (Figure 1B). Moreover, this correlation was significant for female (*r* = −0.70; *p* = 0.03), but not male (*r* = −0.29; *p* = 0.45), subjects. Among the subject characteristics evaluated (Table 1), syncytin-1 expression displayed positive correlation with HbA1c (*r* = 0.58; *p* = 0.01) and negative correlation with HDL-Cholesterol:Total Cholesterol (*r* = −0.47; *p* = 0.05).

### Syncytin-A Expression in Cell Culture Myotubes

Expression of GAPDH in skeletal muscle did not differ between subjects with increased BMI and control subjects (*p* > 0.05). However, ANOVA analysis showed significant effect of cell culture treatments on GAPDH in the myotube experiments (*p* = 0.02). On the other hand, there was no significant effect of cell culture treatments on myosin expression in the myotube epxeriments (*p* = 0.66). Therefore, myosin was used as the housekeeping gene to express responses of syncytin-A and 4E-BP1 in the myotube experiments.

**FIGURE 2:**
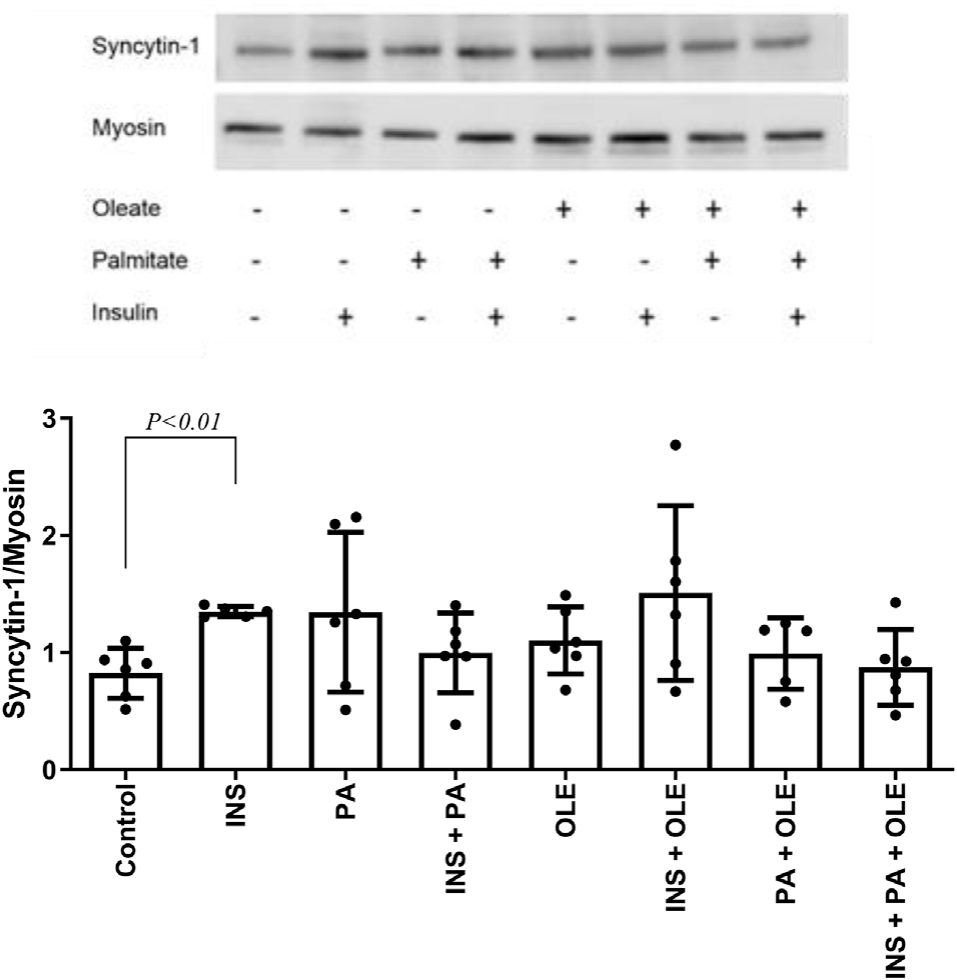
Western blot analysis of syncytin-A expression in differentiated C2C12 myotubes treated with either insulin (INS; 20 nM), palmitate (PA; 300 uM), oleate (OLE; 300 uM), or their combinations, and compared with the no treatment condition (i.e., control). Individual data points are depicted along with mean ± SD.

**FIGURE 3:**
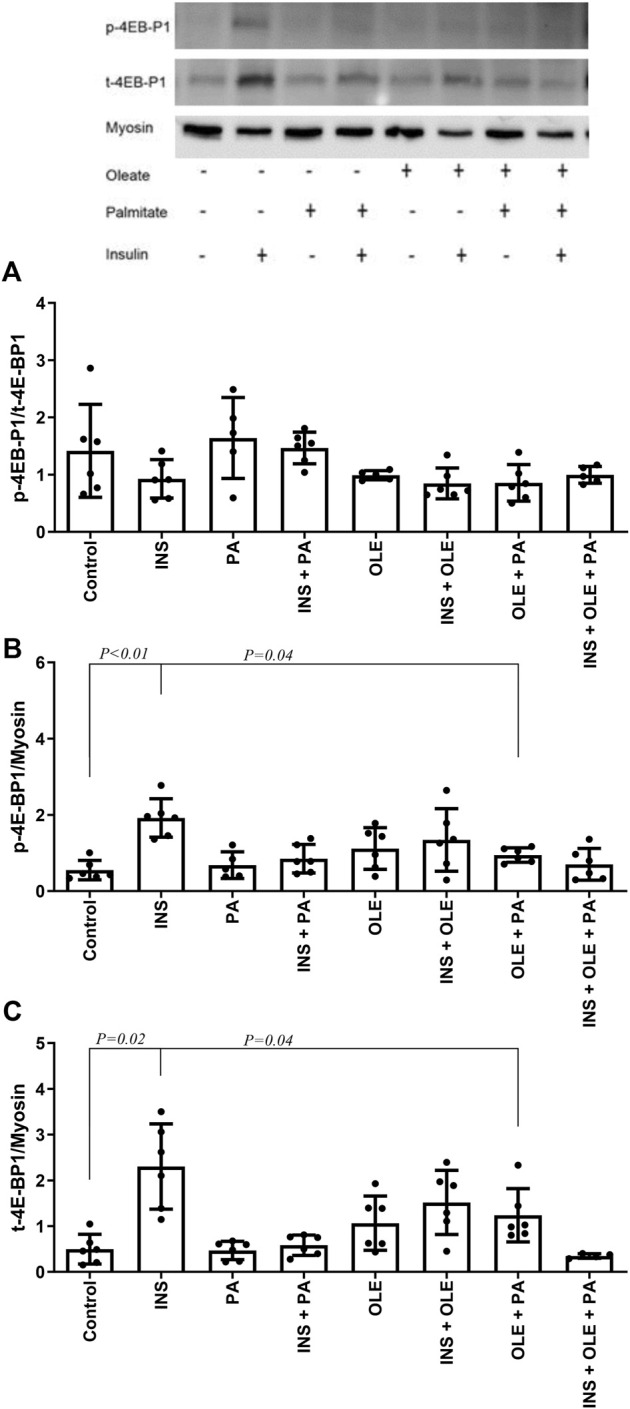
Western blot analysis of p-4E-BP1/t-4E-BP1 **(A)**, p-4E-BP1 **(B)** and t-4E-BP1 **(C)** in differentiated C2C12 myotubes treated with either insulin (INS; 20 nM), palmitate (PA; 300 uM), oleate (OLE; 300 uM), or their combinations, and compared with the no treatment condition (i.e., control). Individual data points are depicted along with mean ± SD.

Insulin treatment alone consistently increased the expression of syncytin-A, but this effect was not significant in the presence of fatty acids (Figure 2). Neither insulin or any of the fatty acid treatments affected the response of p-4E-BP1/t-4E-BP1 (for all *p* > 0.05) (Figure 3A). On the other hand, p-4E-BP1 expression increased by insulin alone, but this effect was not evident when either palmitate or oleate was present along with the insulin (Figure 3B). However, there was a significant effect of combined palmitate and oleate (i.e., without the presence of insulin) on increasing p-4E-BP1 expression (Figure 3B). The effects of insulin and fatty acid treatments on t-4E-BP1 were identical to those for p-4E-BP1 (Figure 3C).

## Discussion

The finding that expression of skeletal muscle syncytin-1 was higher in subjects with BMI in the obese range when compared to subjects with BMI in the lean range was not expected. This is because syncytin-1 regulates muscle maintenance and regeneration/myogenesis (Frese et al., 2015), and these processes are decreased in obesity (Akhmedov and Berdeaux, 2013; O’Leary et al., 2018), and assuming syncytin-1 has a dominant role in upregulating these processes in skeletal muscle. However, there is evidence supporting increase in tissue syncytin-1 expression in pathophysiological circumstances. Syncytin-1 expression is higher in neuronal cells of patients with multiple sclerosis (Antony et al., 2004), a condition characterized by chronic inflammation (Pegoretti et al., 2020), which is also observed in the metabolic environment associated with obesity/increased BMI (Lumeng and Saltiel, 2011). Moreover, treatment of neuronal cells with the inflammatory molecules TNF-α and IL-6, which are also induced within the metabolic environment of obesity (Dorneles et al., 2016; Kern et al., 2018), activates syncytin-1 gene expression (Antony et al., 2007; Mameli et al., 2007). Therefore, it is reasonable to speculate that the inflammatory state linked to obesity may increase the expression of syncytin-1 in skeletal muscle of our subjects characterized by increased BMI. On the other hand, syncytin-1 may induce inflammation through release of NO as well as activation of toll-like receptor family proteins (Wang et al., 2018). Although the exact direction of the cause-effect relationship between syncytin-1 expression and inflammation remains to be determined, the evidence discussed in this paragraph supports increased tissue (i.e., muscle) syncytin-1 expression within the proinflammatory environment associated with increased BMI.

Syncytin-1 is a target gene of the peroxisome-proliferator-activated receptor *γ* (PPAR*γ*), and where stimulation and inhibition of PPAR*γ* increases and decreases syncytin-1 expression, respectively, in human cytotrophoblasts involved in placental development (Ruebner et al., 2012). Therefore, increased syncytin-1 expression in subjects with increased BMI may result from increased muscle PPAR*γ*, whose expression is reported higher in muscle of humans characterized by obesity (Park et al., 1997; Kruszynska et al., 1998). Our novel findings on syncytin-1 in skeletal muscle of humans with increased BMI open the door for future research into the mechanisms implicated, as well as the role and consequences of increased syncytin-1 expression in muscle of these individuals.

Interestingly, the rate of protein synthesis in muscle correlated inversely with the expression of syncytin-1 in muscle. Overexpression of syncytin-1 suppresses its two receptors, alanine/serine/cysteine transporter 1 (ASCT1) and ASCT2 (Antony et al., 2007), two proteins that also serve as cell membrane amino acid transporters (Scopelliti et al., 2013; Scalise et al., 2018). Furthermore, evidence in humans shows that downregulation in the gene expressions of muscle ASCT1 and ASCT2 is observed concurrently with increased expression of syncytin-1 in muscle (Frese et al., 2015). Because reduction in muscle amino acid transporters reduces muscle protein synthesis (Hyde et al., 2005), it is possible that lower protein synthesis in muscle of subjects with increased BMI is observed secondary to a syncytin-1-mediated downregulation of amino acid transport in muscle. In this regard, experimental blockade of these amino acid transport proteins reduces cellular amino acid uptake (Oburoglu et al., 2014). Also, lower protein synthesis in muscle of an animal model of obesity (i.e., Zucker rat) (Reeds et al., 1982) occurs together with lower uptake of amino acids in muscle (Friedman et al., 1990). Therefore, decrease in amino acid transport into muscle secondary to lower content of amino acid transporters in muscle because of higher muscle syncytin-1 expression may explain the inverse correlation we observed between syncytin-1 and protein synthesis in skeletal muscle.

Our cell culture experiments show that insulin stimulates syncytin-A expression in muscle. To our knowledge, our data provide the first evidence describing regulation of this protein by insulin in muscle. Related to this evidence, it is known that syncytin-1 is a target gene for expression through the insulin-like growth factor signaling pathway (Strissel et al., 2008). Our findings suggest that insulin may upregulate muscle growth through increased syncytin-1 expression, and along with effects of insulin on increasing other muscle growth factors, such as myogenin and MyoD (Rochat et al., 2004; Litwiniuk et al., 2016). However, the effect of insulin on syncytin-A expression in the myotube experiments was not evident in the presence of fatty acids, suggesting that fatty acids may interfere with the effects of insulin in increasing the expression of this protein in muscle.

In addition to the effects of insulin on increasing syncytin-A expression, insulin enhanced signaling through the 4E-BP1 pathway in the C2C12 myotubes. This is in line with previous studies showing that insulin activates 4E-BP1 in C2C12 myotubes, and where this effect is observed together with stimulation of protein synthesis (Williamson et al., 2005). Moreover, presence of fatty acids, and specifically palmitate, impairs 4E-BP1 activation in myotubes (Kwon and Querfurth, 2015) and reduces protein synthesis (Perry et al., 2018). Although palmitate did not reduce basal 4E-BP1 signaling in our C2C12 myotube experiments, stimulation of 4E-BP1 by insulin did not occur in the presence of either palmitate or oleate. It has been previously shown that the presence of oleate reverses impaired activation of 4E-BP1 by palmitate (Kwon and Querfurth, 2015) and preserves protein synthesis (Tardif et al., 2014) in C2C12 myotubes. In our cell culture experiments, presence of oleate along palmitate in the cell culture medium enhanced 4E-BP1 signaling. We were not able to evaluate 4E-BP1 signaling in our human subject experiments. However, relevant evidence shows that humans with increased BMI and concomitant reduction in muscle protein synthesis, a subject population comparable to that with increased BMI in our study, display impaired 4E-BP1 expression (Bak et al., 2016).

Concurrent upregulation of syncytin-A and 4E-BP1 expressions by insulin in our cell culture experiments indicates an integrated response with respect to signaling within muscle of processes that under normal physiological circumstances mediate overall myogenesis and protein synthesis. However, these responses may be disassociated in the human metabolic environment associated with increased BMI. Our overall findings constitute original evidence regarding the coordination of biological processes regulating skeletal muscle growth, and how these processes may be differentially affected in humans with increased BMI.

## Conclusion

When compared to controls, humans characterized by increased BMI and concurrent reduction in protein synthesis in skeletal muscle have increased expression of syncytin-1 in muscle. Syncytin-1 expression in muscle shows significant inverse correlation with protein synthesis in muscle. The underlying mechanisms inducing increased expression of syncytin-1 in skeletal muscle of humans with increased BMI and the biological links between syncytin-1 expression and protein synthesis in skeletal muscle deserve further investigation.

## Data Availability

The original contributions presented in the study are included in the article/Supplementary Material, further inquiries can be directed to the corresponding author.
