# Supplementary material for: Increased Expression of Syncytin-1 in Skeletal Muscle of Humans With Increased Body Mass Index
Source: Front Physiol. 2022 Apr 4;13:858341. doi: 10.3389/fphys.2022.858341 (PMC9013906; doi:10.3389/fphys.2022.858341)
Supplement: Supplementary file 1 [file Table1.pdf]

Supplemental Table 1. Circulating non-esterified fatty acids (uM).

		BMI < 25 kg/m^2	BMI > 30 kg/m^2
Myristic	14:0	7.8 ± 4.5	5.7 ± 1.7
Palmitic	16:0	138.1 ± 407	115.1 ± 41.4
Palmitoleic	16:1 cis	15.3 ± 8.5	10.4 ± 3.2
Stearic	18:0	49.8 ± 11.6	51.5 ± 20.6
Oleic	18:1 cis	200.5 ± 67.9	138.2 ± 46.7*
Elaidic	18:1 trans	6.2 ± 3.6	5.1 ± 2.7
Linoleic	18:2 (n-6)	103.1 ± 31.4	79.5 ± 29.5
Linolenic	18:3 (n-3)	9.8 ± 3.3	7.3 ± 2.7
Arachidonic	20:4 (n-6)	3.2 ± 1.0	2.9 ± 1.7
Total		533.9 ± 159.7	415.7 ± 140.7
Oleic/Palmitic		1.44 ± 0.14	1.21± 0.11†

Data are means ± SD; *P<0.05, †P<0.01, versus subjects with BMI < 25 kg/m^2.
